# Rapid Induction of Pulmonary Inflammation, Autoimmune Gene Expression, and Ectopic Lymphoid Neogenesis Following Acute Silica Exposure in Lupus-Prone Mice

**DOI:** 10.3389/fimmu.2021.635138

**Published:** 2021-02-23

**Authors:** Preeti S. Chauhan, James G. Wagner, Abby D. Benninghoff, Ryan P. Lewandowski, Olivia K. Favor, Kathryn A. Wierenga, Kristen N. Gilley, Elizabeth A. Ross, Jack R. Harkema, James J. Pestka

**Affiliations:** ^1^Department of Food Science and Human Nutrition, Michigan State University, East Lansing, MI, United States; ^2^Institute for Integrative Toxicology, Michigan State University, East Lansing, MI, United States; ^3^Department of Pathobiology and Diagnostic Investigation, Michigan State University, East Lansing, MI, United States; ^4^Department of Animal, Dairy and Veterinary Sciences, School of Veterinary Medicine, Utah State University, Logan, UT, United States; ^5^Department of Pharmacology and Toxicology, Michigan State University, East Lansing, MI, United States; ^6^Department of Biochemistry and Molecular Biology, Michigan State University, East Lansing, MI, United States; ^7^Department of Microbiology and Molecular Genetics, East Lansing, MI, United States

**Keywords:** systemic lupus erythematosus, crystalline silica, autoimmune disease, chemokines, type I interferon, B cell, T cell, lung pathology

## Abstract

Occupational exposure to crystalline silica (cSiO_2_) is etiologically associated with systemic lupus erythematosus (lupus) and other autoimmune diseases. cSiO_2_'s autoimmune effects in humans can be mimicked chronically in female lupus-prone NZBWF1 mice following repeated exposure to the particle. However, the immediate and short-term effects of cSiO_2_ in this widely used model of autoimmune disease are not well-understood. In the present study, we tested the hypothesis that a single acute cSiO_2_ dose triggers early presentation of cellular, histopathological, transcriptomic, and protein biomarkers of inflammation and autoimmunity in lupus-prone mice. Eight-week old female NZBWF1 mice were intranasally instilled once with 2.5 mg cSiO_2_ or saline vehicle and necropsied at 1, 7, 14, 21, and 28 d post-instillation (PI). Analyses of bronchoalveolar lavage fluid (BALF) and lung tissue revealed that by 7 d PI, acute cSiO_2_ exposure persistently provoked: (i) robust recruitment of macrophages, neutrophils, and lymphocytes into the alveoli, (ii) cell death as reflected by increased protein, double-stranded DNA, and lactate dehydrogenase activity, (iii) elevated secretion of the cytokines IL-1α, IL-1β, IL-18, TNF-α, IL-6, MCP-1, and B cell activation factor (BAFF), and (iv) upregulation of genes associated with chemokines, proinflammatory cytokines, lymphocyte activation, and type I interferon signaling. The appearance of these endpoints was subsequently followed by the emergence in the lung of organized CD3^+^ T cells (14 d PI) and CD45R^+^ B cells (21 d PI) that were indicative of ectopic lymphoid structure (ELS) development. Taken together, acute cSiO_2_ exposure triggered a rapid onset of autoimmune disease pathogenesis that was heralded in the lung by unresolved inflammation and cell death, proinflammatory cytokine production, chemokine-driven recruitment of leukocytes, an interferon response signature, B and T cell activation, and ELS neogenesis. This short-term murine model provides valuable new insight into potential early mechanisms of cSiO_2_-induced lupus flaring and, furthermore, offers a rapid venue for evaluating interventions against respirable particle-triggered inflammation and autoimmunity.

## Introduction

Systemic lupus erythematosus (lupus) is a devastating autoimmune disease affecting diverse tissues and characterized by intermittent flaring and remission that, over time, can inflict irreversible organ damage (1, 2). Lupus is driven by unresolved inflammation, impaired removal of dead cells, uncovering of autoantigenic epitopes, and aberrant autoantibody responses (3). Resultant autoantigen/antibody complexes deposit in diverse tissues thereby activating the complement system, fostering mononuclear effector cell infiltration, eliciting cytokine/chemokine release, and inducing cell death, which collectively promote tissue destruction and irreversible organ damage. Importantly, autoantigen/autoantibody complex deposition in the kidneys can evoke glomerulonephritis in patients with lupus, often culminating in end-stage renal failure (4).

Multiple genetic, epigenetic, and environmental risk factors contribute to the onset and progression of chronic autoimmune diseases like lupus (5). Examples of environmental factors associated with lupus include cigarette smoking (6, 7), UV radiation (8), and most pertinent to our study, occupational exposure to respirable crystalline silica (cSiO_2_) (9–11). When inhaled, cSiO_2_ particles provoke pronounced recruitment of neutrophils and monocytes/macrophages to the lung, production of proinflammatory mediators, cell death, and fibroblast activation. Incomplete clearance of cSiO_2_ results in chronic inflammation, formation of silicotic nodules, fibrosis, and impaired pulmonary function. In addition to being a human autoimmune trigger, cSiO_2_ is implicated in an array of pulmonary diseases such as silicosis (12), alveolitis (13), pulmonary fibrosis (14), chronic obstructive pulmonary disease (COPD) (15), and lung cancer (16).

In lupus-prone mice, short-term repeated cSiO_2_ exposure mimics lupus flaring by triggering onset and progression of autoimmunity (17, 18). Using the lupus-prone female NZBWF1 mouse model, our laboratory has demonstrated that 4 weekly intranasal instillations of 1 mg cSiO_2_ elicits development of ectopic lymphoid structures (ELS) in the lung, systemic autoimmunity, and early onset of lupus nephritis over the following 3 months post-instillation (PI) (19, 20). In a subsequent study, we determined in the lung how expression of genes associated with immune function are affected by this dosing regimen over a 13-week course of disease progression (21). Transcriptional changes linked to innate and adaptive immune responses were detected within 1 week of the fourth and final cSiO_2_ instillation. Dramatic increases in mRNA transcripts associated with cytokine production, chemokine release, sustained interferon (IFN) activity, complement activation, and adhesion molecules were evident in subsequent weeks. Expression of these genes intensified as disease progressed. Pulmonary transcriptomic signatures were consistent with observed elevations of neutrophils, macrophages, dendritic cells, B cells, and T cells, and collectively, these were consonant with progression of autoimmunity. Taken together, airway exposure to cSiO_2_ in lupus-prone mice evoked aberrant pulmonary transcriptomic signatures for both innate and adaptive immune responses, suggesting that the lung functioned as the nexus for initiating systemic autoimmunity and, ultimately, glomerulonephritis.

While cSiO_2_-triggered human autoimmune flaring can be recapitulated chronically in lupus-prone mice, the short-term responses to this particle in these models are still not well-understood. In this study, we hypothesized that a single bolus cSiO_2_ exposure triggers early presentation of cellular, histopathological, transcriptomic, and protein markers of inflammation and autoimmunity in lupus-prone mice. The specific objective of this study was to determine the immediate and short-term effects (i.e., 1–28 d PI) of a single acute intranasal instillation of 2.5 mg cSiO_2_ on triggering inflammatory markers and autoimmunity in the lungs of female NZBWF1 mice. The results suggested that within 1 week, cSiO_2_ triggered persistent cell death, recruitment of leukocytes, increased proinflammatory cytokine and chemokine expression, and a prominent IFN gene signature in the lung. Remarkably, appearance of these biomarkers was followed within 3 week by the onset of ectopic lymphoid neogenesis.

## Materials and Methods

### Animals

Experimental animal procedures were approved by MSU Institutional Animal Care and Use Committee (AUF #PROTO201800113) in accordance with the guidelines of the National Institute of Health. Six-week-old female NZBWF1 mice were purchased from Jackson Laboratories (Bar Harbor, ME, cat. #100008) and housed 4 animals per cage. This mouse strain was chosen because it has been previously used to recapitulate robust autoimmune triggering by cSiO_2_ that was not seen in normal C57BL/6 mice (19–24). Mice were given free access to drinking water and fed American Institute of Nutrition (AIN)-93G diet (Dyets Inc., Bethlehem, PA) under controlled conditions (humidity: 40–55%; lighting: 12-h light/dark cycles; and temperature: 24 ± 2°C) as described previously (19, 20). Experiments were initiated after 2 weeks of acclimatization.

### Intranasal cSiO_2_ Instillation

Eight-week old NZBWF1 mice were intranasally instilled once with cSiO_2_ as previously described (19). Briefly, cSiO_2_ particles (Min-U-Sil® 5, average particle size: 1.5–2.0 μm, Pennsylvania Sand Glass Corporation, Pittsburgh, PA, US) were acid-washed, oven-dried, and suspended in sterile phosphate buffered saline (PBS; Millipore Sigma). Fresh stock suspensions were prepared prior to use, sonicated, and vortexed for 1 min before intranasal instillation. Mice were anesthetized by inhalation with 4% isoflurane in O_2_, held in the supine position, then instilled once with 1 or 2.5 mg cSiO_2_ in 25 μl PBS or 25 μl of PBS vehicle (Veh). Mice were held in the same position for a short period after instillation to ensure proper distribution inside the respiratory tract. The processing time of the intranasal instillation was <10 s per mouse, and no injury or death occurred during the procedure. In a preliminary study, mice were intranasally instilled with 1 mg cSiO_2_ (*n* = 12) or Veh (*n* = 12). Cohorts of cSiO_2_- and Veh-treated mice (2 groups, *n* = 4) were terminated at days 1, 3, or 7 d post-instillation (PI). For the main study, mice were instilled with either 2.5 mg cSiO_2_ (*n* = 40) or Veh (*n* = 40). Cohorts from cSiO_2_- and Veh-treated mice (2 groups, *n* = 8/group) in the complete study were terminated at days 1, 7, 14, 21, or 28 d PI. The 2.5 mg cSiO_2_ per mouse dose has been widely used to study silicosis (25–30). Doses of 1 and 2.5 mg in mice are allometrically equivalent based on respiration volume of 12 and 30 percent of human lifetime occupational exposure to respirable cSiO_2_, respectively, at the permissible exposure limit (PEL) of 50 μg/m^3^/d established by the U.S. Occupational Safety and Health Administration (31). This PEL is frequently surpassed in occupations related to construction, demolition, mining, and fracking (32, 33).

### Tissue Collection and Preparation

Mice were anesthetized by intraperitoneal injection with 56 mg/kg body weight sodium pentobarbital then euthanized by exsanguination via the abdominal aorta. Bronchoalveolar lavage fluid (BALF) was collected from the lungs as described previously (34). Briefly, after death, the trachea was exposed and cannulated. Heart and lungs were excised *en bloc*. Sterile saline (0.8 ml) was instilled through the cannulated trachea to recover BALF. For each mouse, this procedure was repeated twice and BALF fractions combined for subsequent cell and protein analyses. The caudal lung lobe was then removed and stored in RNAlater (Thermo Fisher Scientific, Wilmington, DE) at −80°C for RNA analysis. Finally, the left lung lobe was intratracheally fixed with 10% (v/v) neutral-buffered formalin (Fisher Scientific, Pittsburgh, PA) at a constant pressure (30 cm H_2_O) for 2 h then immersed and stored in a large volume of formalin fixative for 24 h. After fixation, the left lung lobe was processed in 30% ethanol for histological preparation and further microscopic analysis.

### BALF Inflammatory Cell Quantitation

Total cell counts in BALF were determined by standard counting on a hemocytometer. Cytospin slides were prepared by centrifuging 150 μL of BALF from each mouse at 400 × g for 10 min. Slides were allowed to dry overnight at 25°C and were then stained with Diff-Quick (ThermoFisher). Differential cell counts for macrophages/monocytes, neutrophils, and lymphocytes in BALF were performed based on morphological criteria from a total of 200 cells present on the slides.

### Cell Death Measurement

Cell damage and death were assessed by measuring release of protein, dsDNA, and lactate dehydrogenase (LDH) in BALF. Total protein concentration, an indicator of epithelial and cell membrane integrity, was quantified using a Pierce™ BCA Protein Assay Kit (ThermoFisher, cat.# 23225). Double-stranded DNA, a measure of nuclear damage, was assessed using Quant-iT™ PicoGreen™ dsDNA reagent (Invitrogen, Carlsbad, CA, cat.#P11495) according to the manufacturer's protocol. LDH activity was used to evaluate cytotoxicity and performed as described (35). Briefly, 50 μl of BALF or PBS (blank) was transferred to a 96-well plate, followed by 100 μl of LDH substrate solution (2 mM iodonitrotetrazolium chloride, 3.2 mM β-nicotinamide adenine dinucleotide sodium salt, 160 mM lithium lactate, 15 μM 1-methoxyphenazine methosulfate in 0.2 M Tris-HCl, pH 8.2). Plates were incubated in the dark for 15 min at 25°C then read on a FilterMax F3 Multimode plate reader (Molecular Devices, San Jose, CA) at 492 nm.

### Enzyme-Linked Immunosorbent Assay (ELISA) of Cytokines in BALF

ELISA DuoSets (R&D Systems, Minneapolis, MN) were used to measure IL-1β (cat. #DY401), IL-1α (cat. #DY400), IL-6 (cat. #DY406), TNF-α (cat. #MTA00B), IL-18 (cat. #DY122-05), MCP-1 (cat. #MJE00B), and BAFF (cat. #MBLYS0) in the BALF according to the manufacturer's instructions.

### Lung Histopathology, Immunohistochemistry, and Morphometry

Formalin-fixed left lung lobes were routinely processed and embedded in paraffin. Serial sections of tissue (5 μm) were deparaffinized and stained with hematoxylin and eosin (H&E). H&E stained sections were used for brightfield microscopic imaging and semi-quantitative scoring for the following lung lesions: (a) presence of centriacinar inflammation, (b) presence of centriacinar epithelial hyperplasia, (c) presence of alveolar proteinosis, and (d) presence of perivascular lymphoid cells. Individual lungs were semi-quantitatively graded for these lesions as percent of total pulmonary tissue examined based on the following criteria: (0) no changes compared to control mice; (1) minimal (<10%); (2) slight (10–25%); (3) moderate (26–50%); (4) severe (51–75%); or (5) very severe (>75%) of total area affected. Microscopic and semiquantitative scoring of lung lesions were performed by a certified veterinary pathologist without knowledge of individual animal exposure history.

Lung sections were stained with mouse-specific monoclonal antibody to CD45R (Becton Dickinson, Franklin Lakes, NJ; cat.# 550286) or mouse-specific polyclonal antibody to CD3 (Abcam, Cambridge, MA; cat.#ab5690) as described previously to identify B and T lymphocytes, respectively, which are indicative of ELS. Tissue morphometry was carried out as previously reported (19). Slides were first digitized with a VS110 Virtual Slide System (Olympus). The whole tissue section was chosen as the region of interest and 20% of the sections were captured using systematic random sampling with NewCast software (Visiopharm, Hoersholm, Denmark) and 20X virtual magnification, to yield at least 100 images for morphometric evaluation. Percent of CD45R^+^ and CD3^+^ lung tissue were calculated by projecting a point grid over randomly sampled images with the STEPanizer v1.8 stereology tool and totaling the number of points falling onto positive staining or reference tissue.

### NanoString Autoimmune Gene Profiling

RNA was extracted from lungs of 7, 14, 21, and 28 d PI mouse groups using TissueLyser II (Qiagen) and Tri Reagent (Sigma Aldrich, St. Louis, MO). Extracted RNA was purified using a Zymo RNA Clean and Concentrator Kit with DNase (Zymo Research, Irvine, CA, cat. #R1017). RNA was dissolved in nuclease-free water and quantified using the Qubit (Thermo Fisher Scientific). RNA integrity was also assessed using the Bioanalyzer (Agilent Technologies) at the MSU Genomics Core. Equal amounts of RNA from individual samples within each group were pooled to yield 8 samples for autoimmune profiling across all time points (7, 14, 21, and 28 d PI). Samples (RNA integrity >8) were analyzed using Nano String Autoimmune Gene Expression assay (XT-CSO-MAIP1-12) at the MSU Genomics Core.

Data were processed using nSolver 4.0. Background subtraction was performed using the negative controls included with the module. Background-subtracted samples were normalized using internal positive controls and housekeeping genes included in the module. For hierarchical clustering and heatmap generation of NanoString data, raw counts were first normalized by nSolver analysis per NanoString gene expression data analysis guidelines. Log_2_ transformed normalized counts were used for Heatmap and PCA plot generation using ClustVis [https://biit.cs.ut.ee/clustvis/; (36)]. nSolver 4.0 was further used to analyze differentially expressed genes (DEGs). Genes with log_2_ FC values ≥0.58 (a fold change >50%) were identified as upregulated DEGs and genes with log_2_ FC ≤-0.58 were identified as downregulated DEGs. Statistical analysis of gene expression determined by NanoString was not performed as pooled RNA samples were used. However, expression profiles for 27 genes of interest were verified by qRT-PCR as described below. Venny v2.1 (37) was used to create Venn diagram of DEGs of cSiO_2_-exposed groups at different times as described previously (21).

### Gene Ontology and Network Analysis

Upregulated DEGs identified in common for 7, 14, 21, and 28 d PI were subjected to gene ontology (GO) analysis using Enrichr [https://amp.pharm.mssm.edu/Enrichr/; (38, 39)]. Protein-protein interactions (PPI) among these genes were predicted using STRING (https://string-db.org/) which provides a critical assessment and integration of PPIs from multiple resources, including direct (physical) as well as indirect (functional) associations (40). Interactions among upregulated DEGs were considered with the highest confidence level for associations set at >0.7 and the Markov Cluster (MCL) algorithm inflation parameter set at 3 to identify clusters.

### Quantitative Reverse Transcription Polymerase Chain Reaction (RT-PCR)

RNA was reverse transcribed to cDNA at 100 ng/μl with a High Capacity cDNA Reverse Transcription Kit (Thermo Fisher Scientific, Waltham, MA). TaqMan assays (Thermo Fisher Scientific) were then performed on a Smart Chip Real-Time PCR System with technical triplicates for selected genes of interest and housekeeping genes (*Actb, Gapdh*, and *Hprt*) at the MSU Genomics Core. Gene expression levels were normalized to the endogenous housekeeping genes and reported as fold change relative to the experimental control group using the 2^−ΔΔCT^ method (41).

### Statistics

All statistical analyses except nCounter data were performed using GraphPad Prism version 8 for Windows (GraphPad Software, La Jolla California USA, www.graphpad.com). Data were inspected using the Grubb's outlier test (with *Q* = 1%) to identify potential outliers. Data that did not meet the normality assumption as determined by the Shapiro-Wilk test (*p* < 0.01) were analyzed using the Kruskal-Wallis non-parametric test with Dunn's *post-hoc* test. Data meeting both normality and variance assumptions were analyzed using a two-way analysis of variance (ANOVA) with Sidak's *post-hoc* test (comparisons of treatments at each time point) and Tukey's *post-hoc* test (comparisons across timepoints within treatment groups) as appropriate. Data are presented as mean ± standard error of the mean (SEM). A *p* ≤ 0.05 was considered statistically significant.

## Results

### Acute Dosing With 1 mg cSiO_2_ Causes Modest and Transient Lung Inflammation

**Figure 1:**
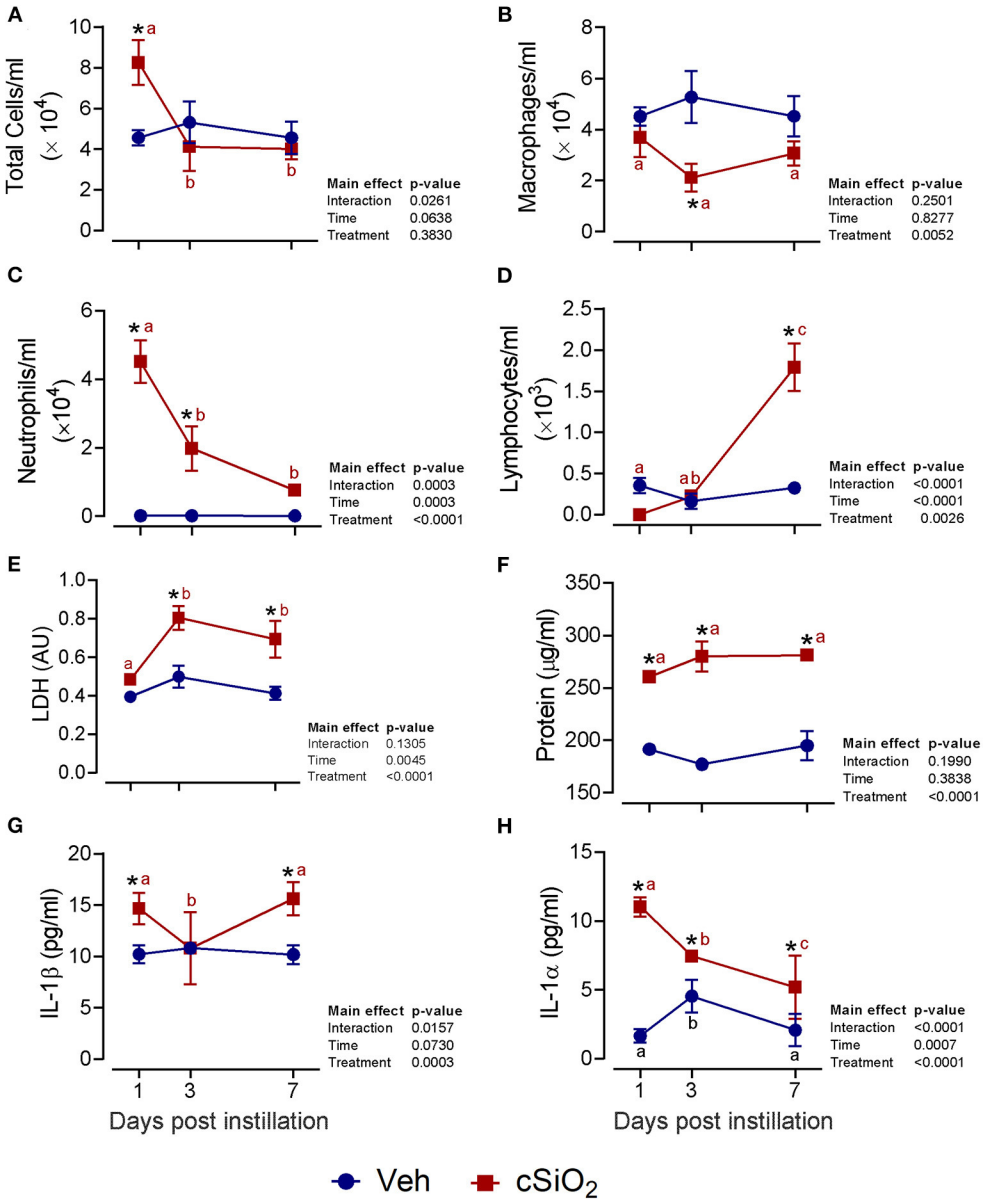
Intranasal instillation with 1 mg cSiO_2_ induces modest lung inflammation in NZBWF1 mice. At 8 week of age, mice were intranasally instilled with 25 μl of PBS vehicle (Veh) or 1 mg cSiO_2_ suspended in 25 μl Veh. Mice were sacrificed at 1, 3, and 7 d post-instillation (PI) and bronchoalveolar lavage fluid (BALF) removed. Inflammatory cell recruitment in BALF was assessed by counting **(A)** total cells, **(B)** macrophages, **(C)** neutrophils, and **(D)** lymphocytes. BALF acellular fraction was analyzed for **(E)** LDH activity and **(F)** protein levels as measures of cell death and for levels of the proinflammatory cytokines **(G)** IL-1β and **(H)** IL-1α. Data are presented as mean ± SEM (*n* = 4). Asterisk indicates significantly different from time-matched Veh control (*p* < 0.05). Values at timepoints within a treatment group without the same letter differ (*p* < 0.05).

The effects of intranasal exposure to 1 mg cSiO_2_ on BALF profiles and lung histopathology in the female NZBWF1 mouse were initially assessed over the course of 7 d (Figure 1). Total leukocyte cell counts were significantly elevated in cSiO_2_-treated mice at 1 d PI but returned to control values by 3 and 7 d PI (Figure 1A). Differential cell counting revealed that macrophage numbers declined in cSiO_2_-treated mice at 3 d PI but recovered by 7 d PI (Figure 1B). Exposure to cSiO_2_ particles at this dose markedly induced neutrophil infiltration 1 d PI, which gradually diminished by 7 d PI (Figure 1C). BALF lymphocyte counts were elevated by cSiO_2_ treatment at 7 d PI (Figure 1D). Consistent with BALF cell counts, cSiO_2_ instillation at 1 mg moderately increased LDH activity (3 and 7 d PI; Figure 1E), total protein concentration (1, 3, and 7 d PI; Figure 1F), IL-1β (1 and 7 d PI; Figure 1G), and IL-1α (1, 3, and 7 d PI; Figure 1H) in the BALF. Only minimal pulmonary inflammation was observed in H&E stained lungs (data not shown). Morphometric scoring for lung lesions showed a minimal score for centriacinar inflammation and no evidence of centriacinar epithelial hyperplasia, proteinosis, or perivascular lymphocyte cells (data not shown) in mice instilled with 1 mg cSiO_2_. Accordingly, instillation with 1 mg cSiO_2_ had modest and transient effects with respect to inflammation in the lung.

### Acute Dosing With 2.5 mg cSiO_2_ Elicits Robust and Persistent Lung Inflammation

**Figure 2:**
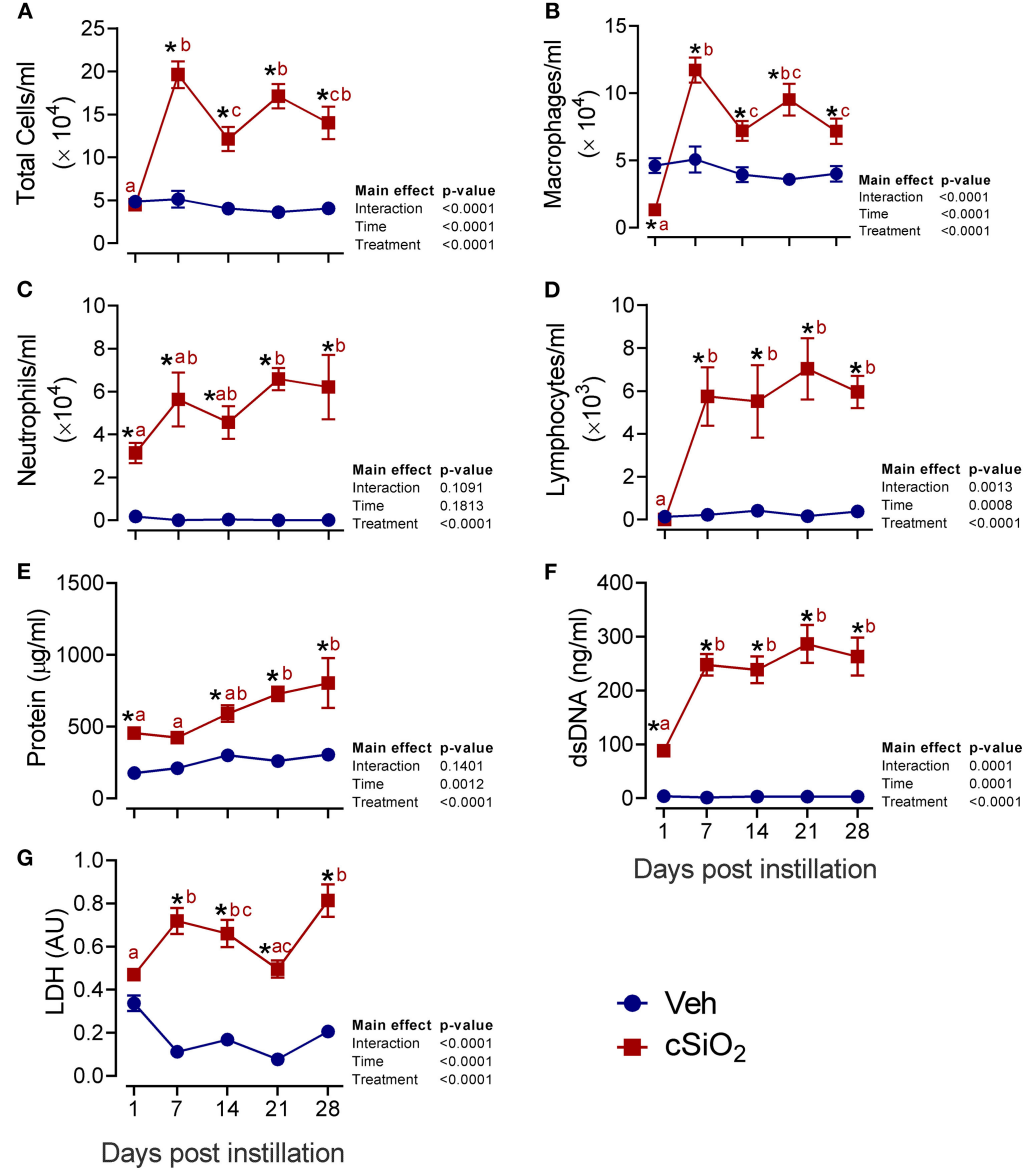
Intranasal instillation with 2.5 mg cSiO_2_ dose elicits intense prolonged inflammatory cell recruitment and cell death in lungs of NZBWF1 mice. Mice were exposed to 2.5 mg cSiO_2_ or PBS vehicle (Veh) by intranasal instillation, sacrificed after 1, 7, 14, 21, and 28 d PI, and BALF removed. BALF cell fraction was assessed for **(A)** total cells, **(B)** macrophages, **(C)** neutrophils, and **(D)** lymphocytes as measures of inflammatory cell recruitment. Acellular BALF fraction was analyzed for **(E)** protein levels, **(F)** dsDNA, and **(G)** LDH activity as measures of cell death. Asterisk indicates significantly different from time-matched Veh control (*p* < 0.05). Values at timepoints within a treatment group without the same letter differ (*p* < 0.05).

**Figure 3:**
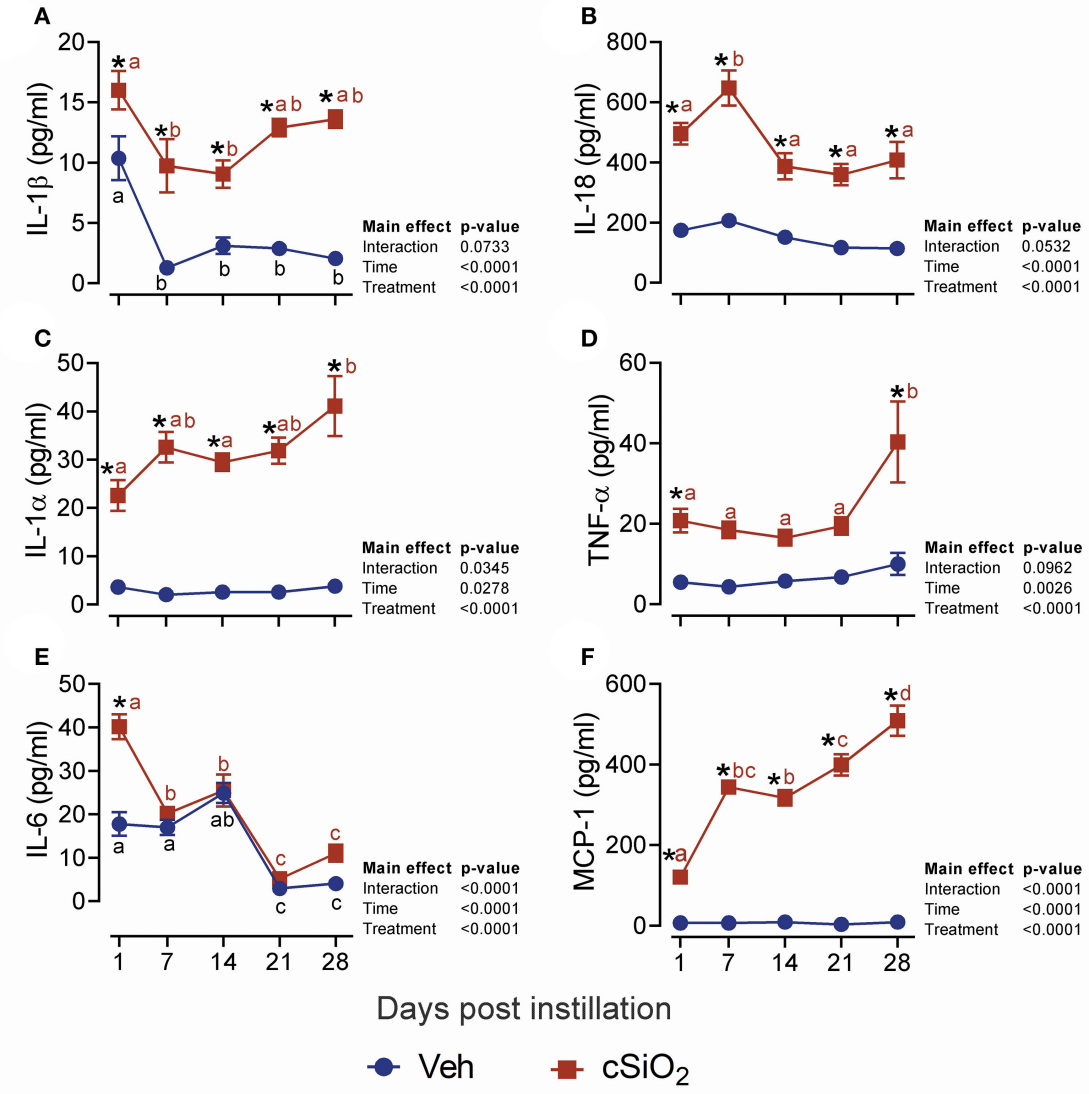
Dosing with 2.5 mg cSiO_2_ induces secretion of proinflammatory cytokines into BALF of NZBWF1 mice. Mice were exposed to Veh or 2.5 mg cSiO_2_ and BALF removed as described in Figure 2 legend. BALF was analyzed by ELISA for: **(A)** IL-β, **(B)** IL-18, **(C)** IL-1α, **(D)** TNF-α, **(E)** IL-6, and **(F)** MCP-1/(CCL2). Data are presented as mean ± SEM (*n* = 8). Asterisk indicates significantly different from time-matched Veh control (*p* < 0.05). Values at timepoints within a treatment group without the same letter differ (*p* < 0.05).

We next evaluated the effects of 2.5 mg cSiO_2_ on female NZBWF1 mice based on wide usage of this dose for investigation of silicosis in non-autoimmune prone mice (25–30). Unlike the 1 mg dose, intranasal instillation with 2.5 mg cSiO_2_ elicited a persistent increase in total BALF leukocytes (Figure 2A), macrophages (Figure 2B), neutrophils (Figure 2C), and lymphocytes (Figure 2D) detectable at 7 d PI and lasting through 28 d PI. While there was an initial decline in macrophages at 1 d PI, the effect was transient and subsequent infiltration by this population represented much of the increase in total cell counts in the BALF, followed by neutrophils and lymphocytes. Correspondingly, the 2.5 mg dose rapidly elicited increases in cell death markers including total protein (Figure 2E), dsDNA (Figure 2F), and LDH activity (Figure 2G), which persisted up to 28 d PI. Concurrent with changes in BALF cell profiles, treatment with 2.5 mg cSiO_2_ induced secretion of proinflammatory cytokines including IL-1β (2- to 7-fold; Figure 3A), IL-18 (2- to 3-fold; Figure 3B), IL-1α (3- to 11-fold; Figure 3C), and TNF-α (3- to 10-fold; Figure 3D) into BALF from 1 to 28 d PI. IL-6 appeared significantly elevated only at 1 d PI (2-fold; Figure 3E). Lastly, chemokine MCP-1 (Figure 3F) concentrations increased in cSiO_2_-treated mice compared to controls throughout the 28 d period from 18-fold at 1 d PI to 56-fold at 28 d PI.

**Figure 4:**
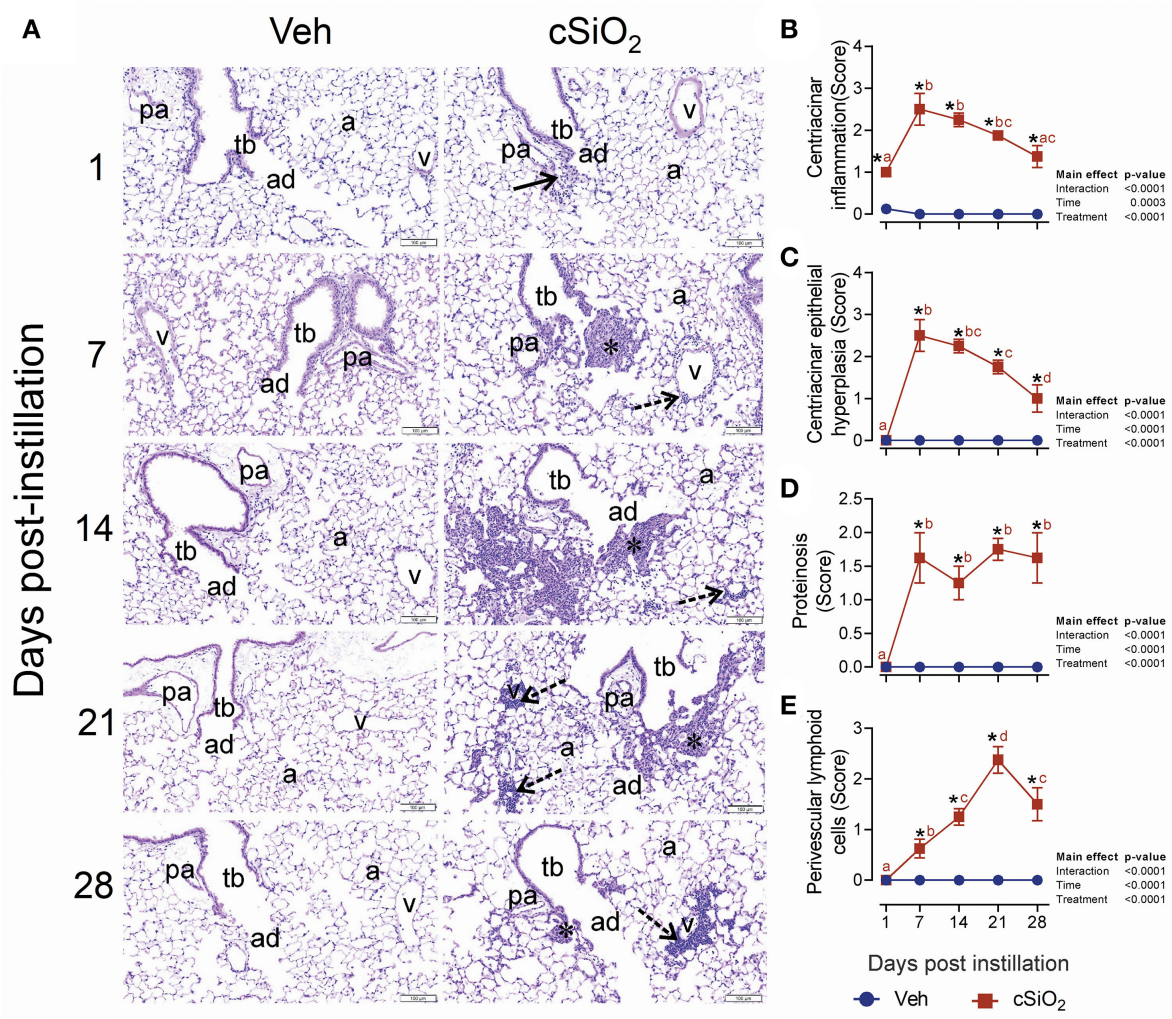
Intranasal instillation with 2.5 mg cSiO_2_ induces persistent lung inflammation in NZBWF1 mice. **(A)** Representative light photomicrographs of hematoxylin and eosin-stained lung tissues (centriacinar regions) from mice intranasally instilled with Veh or 2.5 mg cSiO_2_ and sacrificed after 1, 7, 14, 21, and 28 d PI. Acute, neutrophilic inflammation was present in the centriacinar regions (solid arrows) after 1 d PI in cSiO_2_-treated mice. Centriacinar granulomatous inflammation (foci composed of macrophages, hyperplastic epithelial cells, neutrophils, and lymphocytes; asterisks) was present in cSiO_2_ exposed mice after 7 and 14 d PI but waned in severity by 21 and 28 d PI. Perivascular infiltration of lymphocytes, especially around pulmonary veins (v; stippled arrows) increased in severity from 7 to 28 d after cSiO_2_ exposure, especially at 28 d PI. pa, pulmonary artery; tb, terminal bronchiole; ad, alveolar duct; a, alveolar parenchyma; v, pulmonary vein. Semi-quantitative scores for: presence of **(B)** centriacinar inflammation, **(C)** centriacinar epithelial hyperplasia, **(D)** alveolar proteinosis, and **(E)** perivascular lymphoid cells. Scoring was as follows: 0—no significant finding, 1—minimal, 2—mild, 3—moderate, 4—marked, 5—severe. Data are presented as mean ± SEM (*n* = 8). Asterisk indicates significantly different from time-matched Veh control (*p* < 0.05).

In accordance with elevated leukocyte counts, evidence of cell death, and release of protein mediators in BALF, histologic evaluation in lungs from mice treated with 2.5 mg cSiO_2_ confirmed that extensive inflammation occurred over the 28-d time period (Figure 4A). Acute neutrophilic inflammation was observed in the centriacinar region at 1 d PI. Centriacinar granulomatous inflammation (foci composed of macrophages, hyperplastic epithelial cells, neutrophils, and lymphocytes) was present in cSiO_2_-treated mice at 7 and 14 d PI but lessened in severity by 21 and 28 d PI. Perivascular infiltration of T and B lymphocytes, especially around pulmonary veins, increased in severity throughout the time course but most significantly at 28 d PI. Lung lesions ranged from minimal to mild and included centriacinar inflammation (Figure 4B), centriacinar epithelial hyperplasia (Figure 4C) from 7 to 28 d, PI proteinosis (Figure 4D), and perivascular lymphoid cells from 14 to 28 d PI (Figure 4E).

### Treatment With 2.5 mg cSiO_2_ Induces Rapid and Prolonged Inflammatory and Autoimmune Gene Upregulation

**Figure 5:**
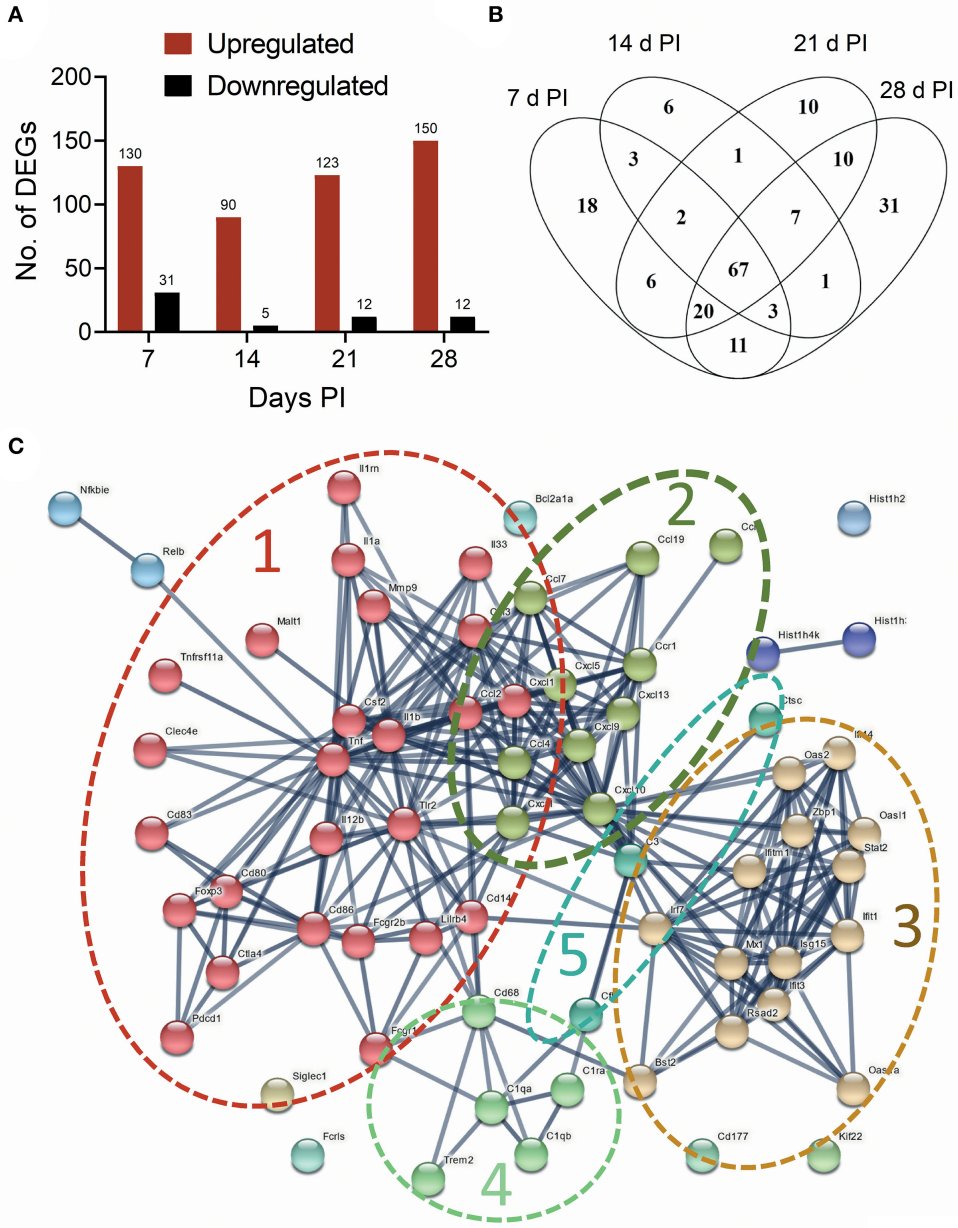
Instillation with 2.5 mg cSiO_2_ induces broad array of genes associated with autoimmunity in the lung after 7, 14, 21, and 28 d. **(A)** nCounter raw data from time course study were subjected to nSolver analysis, and differential gene expression was expressed as log_2_ ratio. Differentially Expressed Genes (DEGs) in lung tissue of mice exposed to cSiO_2_ with respect to Veh-treated controls were selected if they passed the threshold cutoff of log_2_ ratio by 0.58 (≥1.5 FC) for upregulated genes (red bar), and −0.58 (≤1.5 FC) for downregulated genes (black bar). **(B)** Venn diagram illustrating the number of upregulated genes that were shared at 7, 14, 21, and 28 d PI. A total of 67 upregulated genes were differentially expressed at all the time points with respect to Veh control. **(C)** Protein-protein interaction network of commonly upregulated DEGs expressed at all time points created using STRING. The interactions were visualized with a high confidence >0.7. Proteins were clustered using the MCL algorithm. As indicated by dashed lines, several large clusters were identified: cluster 1, proinflammatory genes (red nodes); cluster 2, chemokines (olive nodes); cluster 3, interferon-related genes (tan nodes); clusters 4 and 5, complement (light green and blue nodes, respectively).

The effects of intranasal instillation with 2.5 mg cSiO_2_ on autoimmune transcriptomic signatures were compared at 7, 14, 21, and 28 d PI by NanoString RNA multiplexing. Hierarchical clustering of log_2_ normalized counts exhibited distinct separation of Veh and cSiO_2_ samples at 7, 14, 21, and 28 d PI (Supplementary Figure 1A). Principal component analysis showed clear segregation of the cSiO_2_ samples from Veh samples (Supplementary Figure 1B). Totals of 161, 95, 132, and 162 DEGs were identified at 7, 14, 21, and 28 d PI, respectively. Of these, 130, 90, 123, and 150 genes were upregulated and 31, 5, 12, and 12 genes were downregulated, respectively (Figure 5A). Overall, 67 genes (34.2%) were consistently identified as upregulated DEGs at all time points (Figure 5B and see Supplementary Table 1). No common genes were downregulated across these time points.

A PPI network for the 67 commonly upregulated DEGs was generated using STRING analysis (Supplementary Table 2). The network had a total of 67 nodes (genes) and 288 edges (interactions), which is significantly more interaction than expected from random set of similar size drawn from same genome (32 edges expected from random drawing of genes, enrichment *p* < 1.0e−16). A total of 7 clusters were identified. Based on the number of contained genes, the top five clusters were associated with proinflammatory cytokines, chemokines, lymphocyte activation, interferon signaling, and complement (Figure 5C). Enrichments of GO terms for biological processes identified genes related to cytokine, chemokine, inflammatory, and interferon-mediated responses (Supplementary Figure 2A). GO terms for molecular functions included cytokine, chemokine, and interleukin activity/binding (Supplementary Figure 2B).

**Figure 6:**
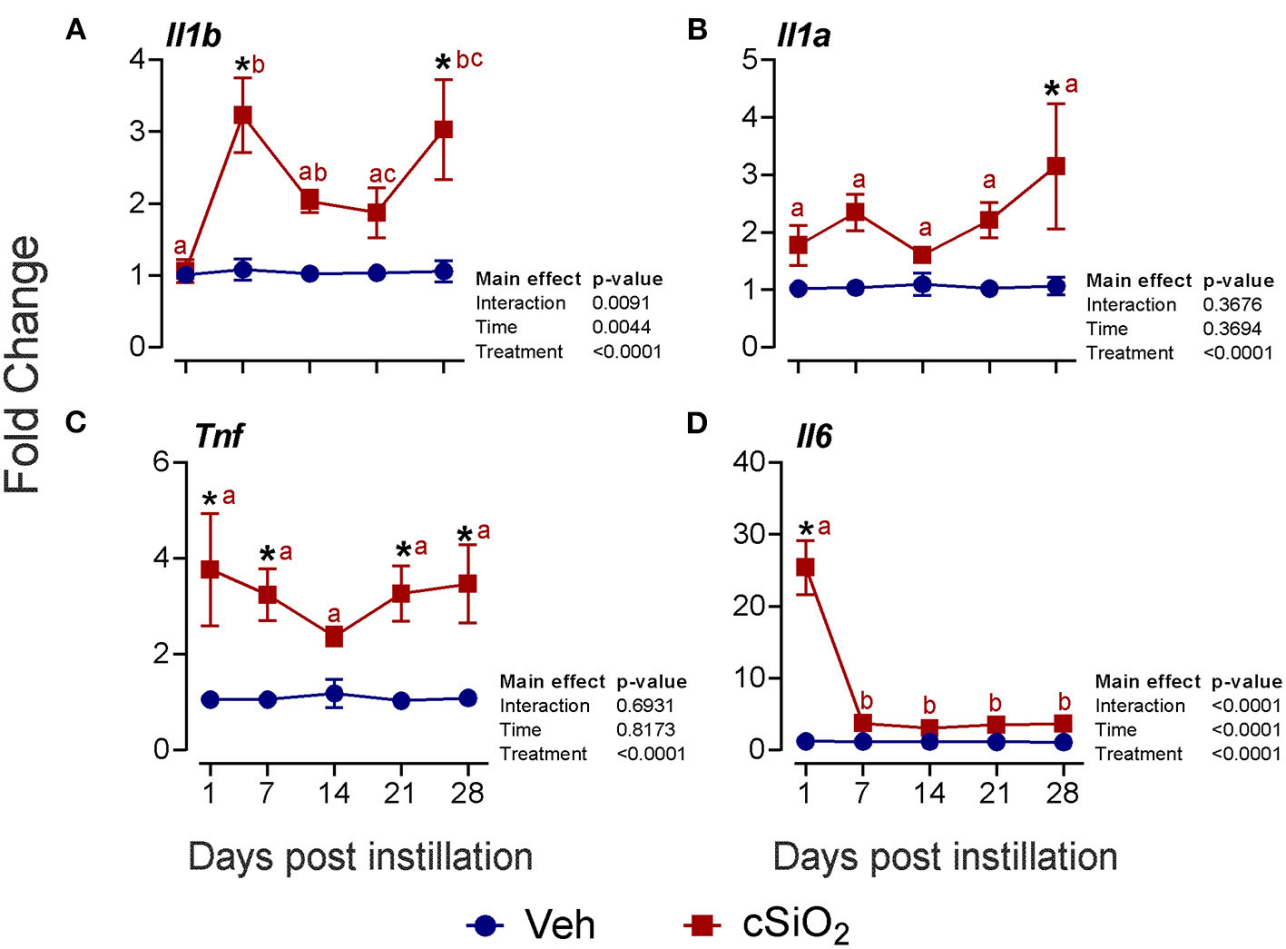
Instillation with 2.5 mg cSiO_2_ induces modest proinflammatory cytokine gene expression in the lungs of NZBWF1 mice. Selected upregulated proinflammatory genes in the lungs were verified by quantitative RT-PCR. Panels show fold change expression of **(A)** *Il1b*, **(B)** *Il1a*, **(C)** *Tnf*, and **(D)** *Il6* between Veh- and cSiO_2_-treated mice. Data are presented as mean ± SEM (*n* = 8). Asterisk indicates significantly different from time-matched Veh control (*p* < 0.05). Values at timepoints within a treatment group without the same letter differ (*p* < 0.05).

Selected mRNAs representing major DEG signatures identified by NanoString multiplexing were quantified by RT-PCR at 1, 7, 14, 21, and 28 d PI. Upregulation of the proinflammatory cytokine genes *Il1b* (2- to 3-fold; Figure 6A), *Il1a* (2- to 3-fold; Figure 6B), *Tnf* (2- to 4-fold; Figure 6C), and *Il6* (3- to 25-fold; Figure 6D) was evident for some or all of the 28-d time period.

**Figure 7:**
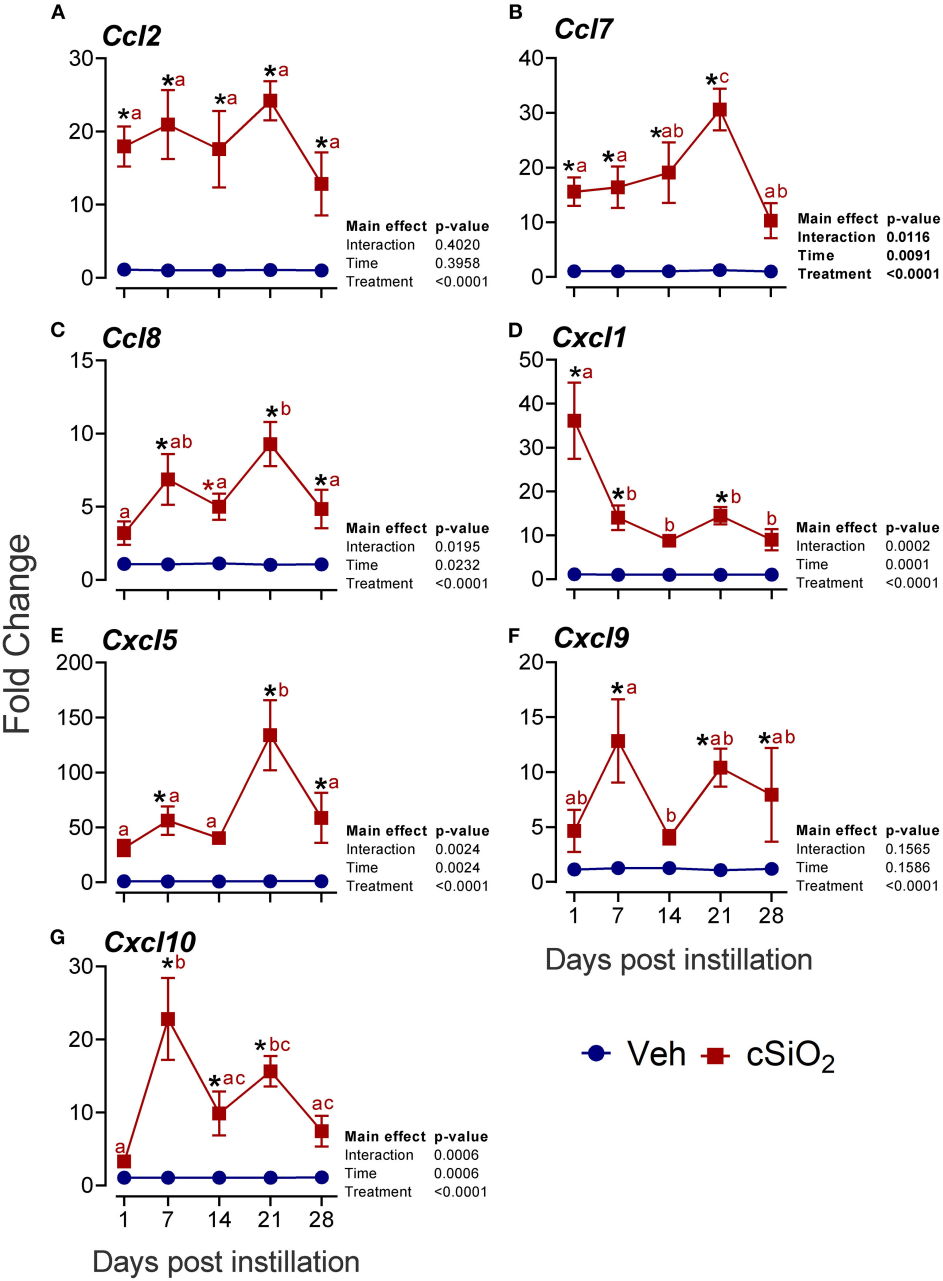
Intranasal exposure to 2.5 mg cSiO_2_ induces rapid and vigorous chemokine mRNA responses in the lungs of NZBWF1 mice. Selected upregulated CC and CXC DEGs were verified by quantitative RT-PCR. Panels show fold change expression of **(A)** *Ccl2*, **(B)** *Ccl7*, **(C)** *Ccl8*, **(D)** *Cxcl1*, **(E)** *Cxcl5*, **(F)** *Cxcl9*, and **(G)** *Cxcl10* between Veh- and cSiO_2_-treated mice. Data are mean ± SEM (*n* = 8). Asterisk indicates significantly different from time-matched Veh control (*p* < 0.05). Values at timepoints within a treatment group without the same letter differ (*p* < 0.05).

More pronounced elevations from 1 to 28 d PI were found for the chemokine-related genes *Ccl2* (13- to 24-fold; Figure 7A), *Ccl7* (10- to 30-fold; Figure 7B), *Ccl8* (4- to 9-fold; Figure 7C), *Cxcl1* (8- to 36-fold; Figure 7D), *Cxcl5* (33- to 134-fold; Figure 7E), *Cxcl9* (4- to 13-fold; Figure 7F), and *Cxcl10* (3- to 23-fold; Figure 7G).

**Figure 8:**
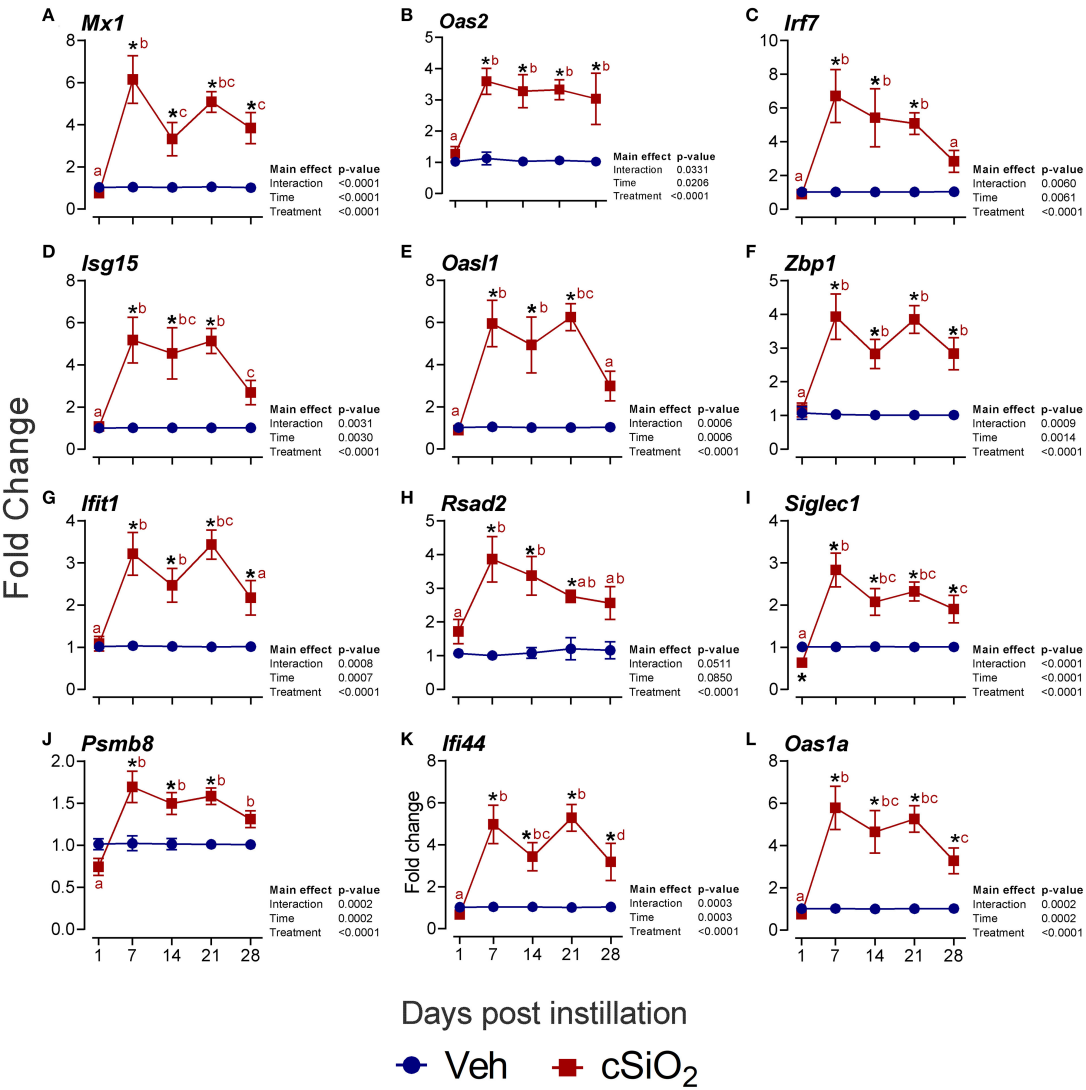
Intranasal instillation with 2.5 mg cSiO_2_ evokes persistent interferon-related gene (IRG) mRNA expression in the lungs of NZBWF1 mice. Selected upregulated IRG DEGs were verified by quantitative RT-PCR. Panels show fold change expression of **(A)** *Mx1*, **(B)** *Oas2*, **(C)** *Irf7*, **(D)** *Isg15*, **(E)** *Oasl1*, **(F)** *Zbp1*, **(G)** *Ifit1*, **(H)** *Rsad2*, **(I)** *Siglec1*, **(J)** *Psmb8*, **(K)** *Ifi44*, and **(L)** *Oas1a*. Data are presented as mean ± SEM (*n* = 8). Asterisk indicates significantly different from time-matched Veh control (*p* < 0.05). Values at timepoints within a treatment group without the same letter differ (*p* < 0.05).

Furthermore, the interferon-related genes *Mx1* (Figure 8A), *Oas2* (Figure 8B), *Irf7* (Figure 8C), *Isg15* (Figure 8D), *Oasl1* (Figure 8E), *Zbp1* (Figure 8F), *Ifit1* (Figure 8G), *Rsad2* (Figure 8H), *Siglec1* (Figure 8I), *Psmb8* (Figure 8J), *Ifi44* (Figure 8K), and *Oas1a* (Figure 8L) were elevated up to 6-fold from 7 to 28 d PI. Accordingly, these quantitative RT-PCR data confirmed key findings generated by NanoString autoimmune profiling of pooled samples.

### Dosing With cSiO_2_ at 2.5 mg Promotes Ectopic Lymphoid Neogenesis in the Lung

**Figure 9:**
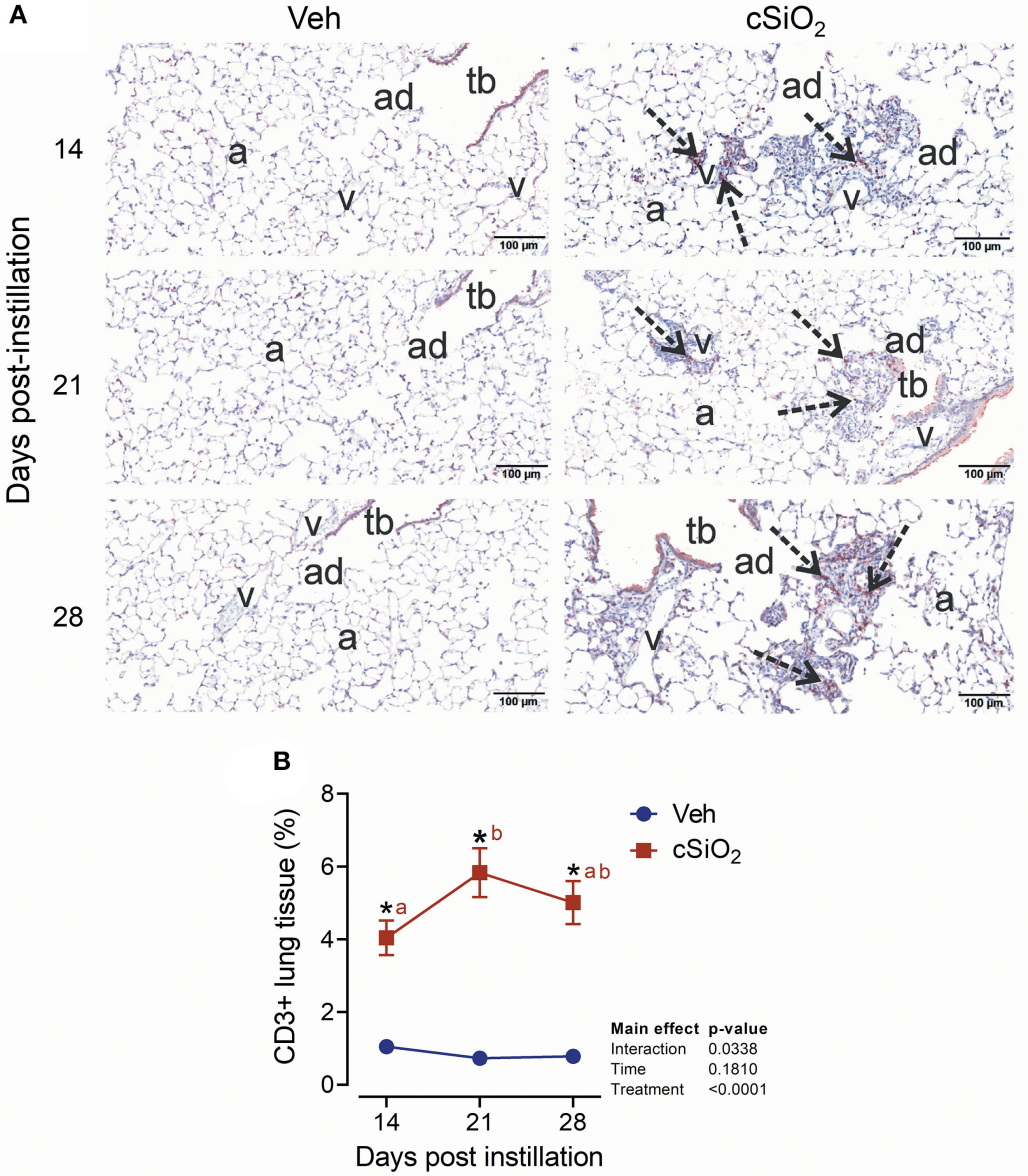
Instillation with 2.5 mg cSiO_2_ causes T cell infiltration into lung tissue of NZBWF1 mice. **(A)** Representative light photomicrographs of lung tissues from mice intranasally instilled with Veh and 2.5 mg cSiO_2_ and sacrificed after 14, 21, and 28 d PI. Lung tissue were stained with anti-CD3 antibody to identify T-lymphocytes and counterstained with H&E. Presence of CD3+ cell infiltration in perivascular interstitium (arrow) induced by cSiO_2_ by 14, 21, and 28 d PI. **(B)** Morphometric quantitation of T cell cellular infiltration in lung parenchyma in mice instilled Veh and cSiO_2_. Data are mean ± SEM (*n* = 8). Asterisk indicates significantly different from time-matched Veh control (*p* < 0.05). Values at timepoints within a treatment group without the same letter differ (*p* < 0.05).

**Figure 10:**
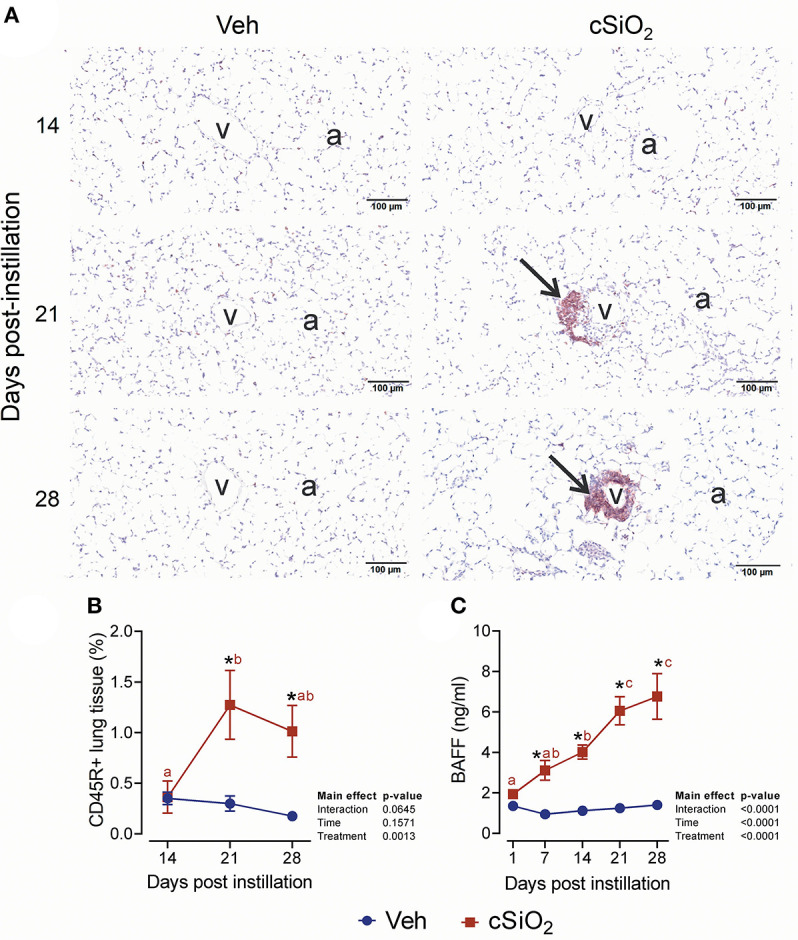
Exposure to 2.5 mg cSiO_2_ stimulates B cell infiltration into lungs of NZBWF1 mice. **(A)** Representative light photomicrographs of lung tissue from mice intranasally instilled with Veh or 2.5 mg cSiO_2_ and sacrificed after 14, 21, and 28 d PI. Lung tissues were stained with anti-CD45 antibody to identify B-lymphocytes and counterstained with H&E. Presence of CD45R+ cell infiltration in perivascular interstitium (arrow) induced by cSiO_2_ by 21 and 28 d. **(B,C)** Morphometric quantitation of B cells. B cells started to accumulate in perivascular region by day 21 and 28 in cSiO_2_ exposed mice. Data are presented as mean ± SEM (*n* = 8). Asterisk indicates significantly different from time-matched Veh control (*p* < 0.05). Values at timepoints within a treatment group without the same letter differ (*p* < 0.05).

The effect of 2.5 mg cSiO_2_ on ELS development was evaluated by immunohistochemical staining. Using anti-CD3 antibody, elevated T cells were detected in the perivascular region of the lung in cSiO_2_-treated mice (Figure 9A). T cell elevation was verified by morphometrically counting the total lung percentage at 14, 21, and 28 d PI (Figure 9B). Upon employing anti-CD45R antibody, B cells were found to be organized around the perivascular areas following cSiO_2_ treatment at 21 and 28 d PI (Figure 10A), which was again morphometrically verified (Figure 10B). Relatedly, the B cell activation factor BAFF (Figure 10C) increased continuously throughout 28 d PI period. Together, these findings suggest that by 21 d PI, ELS commenced development in the lungs of cSiO_2_-treated mice.

## Discussion

This investigation is the first to explore how a single bolus exposure to cSiO_2_, an established human autoimmune trigger, influences the immediate and short-term appearance of biomarkers for autoimmunity onset in a murine model of lupus. Several findings were made that provide new insights into how cSiO_2_ triggers autoimmunity. First, the 2.5 mg cSiO_2_ dose provoked stronger and more persistent recruitment of neutrophils, macrophages, and lymphocytes into the alveoli compared to the 1 mg dose; therefore, the higher dose was used for assessing markers of inflammation and autoimmunity over 28 d. Second, cSiO_2_ instillation rapidly evoked elevation of three cell death indicators in the BALF—protein, dsDNA, and LDH activity—that lasted for the duration of the 28-d study. Third, consistent with pulmonary leukocyte infiltration, cSiO_2_ induced persistent increases in IL-1α, IL-1β, IL-18, TNF-α, IL-6, and MCP-1 in the BALF. Fourth, within the first 7 d PI, cSiO_2_-treated mice exhibited transcriptomic signatures indicative of upregulated expression of genes associated with proinflammatory cytokines, chemokines, lymphocyte activation, type I IFN signaling, and complement that also lasted until the end of the study. Lastly, appearance of cell death, cytokines, and gene biomarkers was closely followed by rising BAFF concentrations in the BALF and the emergence of organized regions of T cells (14 d PI) and B cells (21 d PI) in the lung, indicative of ELS development. Collectively, these observations suggest that acute exposure to cSiO_2_ contributed to fast-developing unresolved inflammation, cell death, and innate immune responses in the lung that were, in turn, followed by adaptive immune responses heralding autoimmunity onset.

While 1 mg cSiO_2_ caused slight induction of total cells in BALF that quickly returned to basal level, the 2.5 mg dose elicited elevated leukocyte counts throughout the 28-d experimental period. These findings for the lower dose of cSiO_2_ corresponded with a lack of histopathologic effects in the lungs of mice treated, whereas the higher dose stimulated rapid and prolonged inflammatory cell recruitment reflected by persistent infiltration of neutrophils, macrophages, and later, lymphocytes. Consistent with our observations, when pulmonary inflammation in C3H/He mice was compared 4 week after intratracheal instillation with 0, 0.375, 0.75, 1.5, 3, 5, and 6 mg cSiO_2_, protein concentration and cell numbers in the BALF increased in linear dose-response fashion and reached a maximum effect at the 3 mg dose (42). Additionally, the heightened cellular inflammatory responses to 2.5 mg cSiO_2_ observed here are in agreement with those seen in many other silicosis studies in non-autoimmune prone mice employing the same dose (25–30). In another relevant study, Chen et al. (43) intratracheally instilled C57Bl/6 mice with 2.5 mg cSiO_2_ and subjected lungs to RNA sequencing after 60 d. Out of 14,451 total genes, 819 genes were found to be differentially expressed with 749 and 70 being upregulated and downregulated, respectively; many of these transcripts were cytokine, chemokine, and interferon-regulated genes. Together, these findings in a variety of mouse strains support the contention that the 2.5 mg cSiO_2_ dose overwhelms normal pulmonary clearance mechanisms and promotes unresolved inflammation, thereby driving an overly robust innate immune response.

Following entry of foreign particles into tissues, neutrophils (44) then monocytes (45) are recruited quickly to the injury site. Infiltration by neutrophils likely is a first critical step in the initiation of autoimmunity in cSiO_2_-exposed NZBWF1 mice. Neutrophils act as the first responders of the innate immune system and are recruited to the injury site, where they become activated. After their activation, they (i) participate in phagocytosis, (ii) undergo degranulation leading to discharge of enzymes, proinflammatory mediators, and reactive oxygen species, (iii) secrete extracellular traps; and finally die by apoptosis or necrosis—all of which contribute to robust inflammation and tissue injury (46–49). Thus, the observation that the high cSiO_2_ dose increased neutrophil numbers beginning at 1 d PI that persisted through 28 d PI is important from the perspective of inflammation and cell death. This persistent presence of neutrophils likely led to further recruitment of monocytes and lymphocytes.

Phagocytosis of cSiO_2_ by macrophages triggers lysosomal membrane permeabilization, inflammasome activation, release of IL-1 family cytokines, and cell death by apoptosis, pyroptosis, or necrosis (50, 51). It was thus noteworthy that there was a reduction of macrophage counts at 1 d PI in the BALF from mice treated with 2.5 mg cSiO_2_, which corresponded to elevated protein, dsDNA, and LDH release. By 7 d PI, macrophage numbers rebounded due to recruitment, exceeding control values at this and all subsequent time points. Macrophage death can further lead to release of free cSiO_2_ particles and cell debris to the surrounding milieu. This continual presence of cSiO_2_ particles elicits repeated cycles of phagocytosis of free cSiO_2_, inflammasome activation, and cell death in macrophages, reemergence of free cSiO_2_, and accumulation of cell debris. cSiO_2_-exposed alveolar macrophages release a large array of cytokines and chemokines (52–55). These signaling molecules can activate other alveolar macrophages in an autocrine fashion and nearby cells (e.g., macrophages, lung epithelial cells) in a paracrine manner to release additional mediators, thereby facilitating further recruitment of inflammatory cells (56).

Particularly notable here were the rapid elevations in IL-1 family cytokines IL-1α, IL-1β, and IL-18. IL-1α is constitutively expressed at high concentrations in alveolar macrophages but is also found in non-hematopoietic cells (57). Considered to be an alarmin, IL-1α is released during lytic cell death and has been deemed to be a master regulator of cSiO_2_-induced acute lung inflammation (55). The observation that cSiO_2_-induced increases in BALF IL-1α protein concentrations were between 6- to 11-fold during the study period, whereas significant IL-1α mRNA elevation (3-fold) was observed only at 28 d PI, is consistent with the possibility that most IL-1α is constitutively produced. Rider et al. (58) demonstrated *in vitro* that IL-1α promotes neutrophil recruitment, whereas mature IL-1β recruits macrophages. In support of this possibility, the authors demonstrated that when C57Bl/6 mice were exposed to 2.5 mg cSiO_2_, robust IL-1α responses were detectable in BALF within 1 h, well before the appearance of neutrophils and elevated IL-1β at 6 h. Alveolar macrophages were the primary source of IL-1α in that study and, furthermore, neutrophil infiltration and IL-1β release were suppressed in IL-1α knockout mice and mice treated with anti-IL-1α antibody.

It is plausible that cSiO_2_-induced IL-1α protein activates the IL-1 receptor 1 on macrophages in an autocrine and paracrine fashion and induces expression of inflammasome components, thereby facilitating cSiO_2_-triggered inflammasome activation with attendant release of active IL-1β and IL-18 (55). IL-1 family cytokines might also drive the increased expression of other proinflammatory cytokines. Relatedly, IL-6 and TNF-α protein levels were elevated in the BALF and the corresponding mRNAs were upregulated in the lungs of cSiO_2_-treated mice within 24 h. Interestingly, while TNF-α mRNA and protein remained elevated for 28 d, IL-6 mRNA and protein returned to control levels by 7 d, suggesting that the latter is under tight transcriptional control (59). Collectively, IL-1 family cytokines, IL-6, and TNF-α could be expected to contribute to the induction of inflammation seen following acute exposure to cSiO_2_ (58, 60–65). Further studies are needed to characterize the centrality of IL-1 family responses of NZBWF1 mice to downstream cytokine responses.

Chemokines play an essential role in selective recruitment of cells during inflammation (66–68). Chronic exposure to cSiO_2_ has been shown to induce expression of a wide range of chemokine genes (21, 43). Likewise, acute cSiO_2_ instillation in the present study induced upregulated expression of chemokines of both the C-C and the C-X-C motifs. Two types of mRNA responses were apparent. The first response was characterized by robust expression at 1 d PI of genes for CCL2 and CCL7 which promote recruitment of monocytes and for CXCL1 which recruits neutrophils. The second group of genes had prominent responses at ≥7 d PI. These included mRNAs for CCL8 (monocyte chemoattraction), CXCL5 (neutrophil chemoattraction and activation), and CXCL9,10 (activated T cell chemoattraction). Accordingly, prolonged and increased expression of these chemokine genes likely leads to increased migration of neutrophils, macrophages, and lymphocytes that were observed here in the BALF and lungs. Consistent with this premise, we observed early appearance of the MCP-1 (i.e., CCL2 protein) in the BALF of cSiO_2_-treated mice which continued to rise throughout the remainder of the experiment. MCP-1 is released predominately by monocytes/macrophages and mediates recruitment of monocytes from systemic circulation to local sites of inflammation after tissue injury (69). Therefore, MCP-1 might be in part responsible for this increase in macrophages observed during d 7 through 28 PI.

IFN signatures are frequently associated with lupus and other autoimmune diseases (70). As observed in chronic studies with NZBWF1 mice (21), IFN-related genes were found to be upregulated in our acute cSiO_2_ model at 1 week PI. Cellular debris released during pyroptosis and necrosis can potentially activate cytosolic receptors that promote an IFN-related gene response. DNA released by lung damage induces a type I IFN response after experimental cSiO_2_ exposure (51). Expression of type I IFN is mediated by dsDNA-driven stimulation of TLR4/7/9 and activation of MyD88 (71–73). Thus, it was interesting in the present study that cSiO_2_ rapidly and persistently induced dsDNA release in BALF that may be responsible for the IFN response.

Sustained inflammatory responses along with impaired clearance of cell debris from neutrophils and macrophages have been associated with the onset of autoimmunity (74). Development of ELS is an established hallmark of autoimmune disease characterized by organized aggregations of B cells, T cells, and follicular dendritic cells (75). ELS behave as tertiary lymphoid organs that act as centers of autoantigen presentation, B cell proliferation and differentiation, and production of autoantibodies (76). Here, we observed that lymphocyte numbers were significantly increased in the BALF from 7 to 28 d PI after cSiO_2_ exposure. Corresponding with increased numbers of BALF lymphocytes, we observed significant accumulation CD3^+^ and CD45R^+^ cells around the perivascular region of the lung reflecting initiated ELS development. These findings are consistent with our previous studies showing that repeated exposures to 1 mg cSiO_2_ triggered inflammation, ELS formation, and AAb production in female NZBWF1 mice (19, 20). Recently, our laboratory has applied high-throughput AAb microarray profiling of 122 autoantigens to ascertain in female NZBWF1 mice how 4 weekly 1 mg cSiO_2_ instillations influence the BALF and plasma AAb repertoire relative to specificity at 1, 5, 9, or 13 week PI of the last dose (24). Marked IgG and IgM AAb responses against lupus-associated autoantigens including ribonucleoprotein, Smith antigen, DNA, histones, Ro/SSA, SSB, and complement by 1 week PI in BALF and 5 week PI in plasma, peaking at 9 and 13 weeks PI, respectively. Furthermore, cSiO_2_ remarkably triggered AAbs linked with ANCA vasculitis, systemic sclerosis, Sjögren's syndrome, rheumatoid arthritis, autoimmune hepatitis, and Hashimoto's thyroiditis. Significantly, cSiO_2_-induced AAb production correlated with accumulation of dead cells in the lung, inflammatory/autoimmune gene expression, and ELS development. Therefore, in the future, it will be important to assess AAb responses in the acute cSiO_2_ model described here.

This study provides valuable new clues into the initial mechanisms that drive cSiO_2_-induced ELS neogenesis. Specifically, cSiO_2_-triggered cytokine, chemokine, and type I IFN expression likely promote leukocyte recruitment and differentiation, which are essential for the development and maintenance of these structures in the lung. Of particular importance here was the continuous increase in the B cell survival factor BAFF in lung alveolar fluid. BAFF, a TNF family member, is expressed by neutrophils, monocytes, macrophages, dendritic cells, and activated T cell subsets (77). BAFF stimulates B cell proliferation and survival in a normal immune response; however, excessive BAFF concentrations can facilitate development of ELS and autoimmunity (78–81). Though we observed continuously increasing BAFF protein concentrations in BALF of cSiO_2_-treated mice, it is interesting to note that NanoString and RT-PCR analyses failed to reveal an analogous effect of cSiO_2_ instillation on BAFF mRNA expression in the lung (data not shown). BAFF is produced as a membrane-bound protein that can be cleaved to a soluble form by furin and other proteases of the pro-protein convertase family (82). It is tempting to speculate that cSiO_2_ treatment induces soluble BAFF concentrations in the lung alveolar fluid by increasing pro-protein convertase expression, but future experiments will be required to investigate this possibility.

A potential limitation of this study was the use of intranasal instillation. This approach was selected because of its use by us and others for triggering autoimmunity in prior chronic studies with lupus-prone NZBWF1 mice (19–21) and NZM2410 mice (18, 83–85). We do however recognize that alternative routes of cSiO_2_ exposure are possible, including intratracheal instillation, oropharyngeal aspiration, and ambient inhalation. When BALB/c mice were subjected to intratracheal, oropharyngeal, and intranasal instillation with cSiO_2_ at 1 mg in a comparative study, all three approaches elicited comparable pulmonary inflammation (86). Regarding ambient inhalation, there is one reported study employing NZBWF1 mice (17) in which animals were exposed to the particle at 70 mg/m^3^ cSiO_2_ for 5 h/d for 12 consecutive days then euthanized 16 week later. Assuming a mouse inhalation rate of 0.03 l/min, this would equate to a maximum lung load of 8 mg. Exposed mice showed intense accumulation of neutrophils, macrophages, and lymphocytes in the BALF as well as peribronchial and perivascular lymphocytic infiltrates and bronchial-associated ELS remarkably like our findings here. Given the similar responses across methods, we believe intranasal exposure as employed here is suitable for recapitulating and elucidating early events during cSiO_2_-triggering autoimmunity in lupus-prone mice.

Another possible limitation of this study was not including normal mice without predilection for autoimmunity for comparative purposes. We chose to do this because prior studies suggest that normal mice do not develop the cSiO_2_-triggered autoimmune responses seen in lupus-prone NZBWF1 mice. For example, when the NZW/LacJ strain, a parental strain for hybrid NZBWF1 that does not spontaneously develop glomerulonephritis (87), was intranasally instilled with 4 weekly 1 mg doses of cSiO_2_ to mimic chronic exposure, it did not exhibit the robust ELS development or nephritis seen in NZBWF1 mice. C57BL/6 mice similarly do not exhibit ELS development when exposed to an identical cSiO_2_ regimen (19). Concordantly, cSiO_2_ inhalation studies suggest that, while NZBWF1 mice exhibit ectopic lymphoid neogenesis, both C3H/He and BALB/c mice are recalcitrant to this response (17). Nevertheless, it should be noted that when C57BL/6 mice are instilled with cSiO_2_ and their lungs assessed 60 d later, similar mRNA signatures to those seen here including upregulated chemokine and interferon-regulated genes are observed (43). Since it is necessary to discriminate magnitude and specificity of aberrant responses in autoimmune mice from those found in normal mice, future studies should include comparison of short-term biomarker responses to cSiO_2_ concurrently in both genotypes.

In conclusion, the present study links cSiO_2_ triggering of unresolved inflammation with early changes in the transcriptome, and, ultimately, autoimmune disease in the female NZBWF1 mouse. Particularly intriguing were the early and persistent effects of cSiO_2_ on IL-1α, chemokines, and BAFF in the lung. This short-term murine model provides valuable new understanding into early mechanisms of cSiO_2_-induced lupus flaring and furthermore, offers a rapid venue for evaluating interventions against particle triggered inflammation and autoimmunity. In the future, it will be critical to distinguish the cells that are responsible for these early mRNA transcript signatures and release of cytokines/chemokines.

## Data Availability Statement

The raw data supporting the conclusions of this article will be made available by the authors, without undue reservation, to any qualified researcher.

## Ethics Statement

This animal study was reviewed and approved by MSU Institutional Animal Care and Use Committee (AUF #PROTO201800113).

## Author Contributions

PC: study design, data analyses/interpretation, figure preparation, manuscript preparation, investigation, and manuscript editing. JW: study design, necropsy, and lab analyses. AB: study design, data analyses, figure preparation, and manuscript writing. RL: necropsy and lab analyses. OF and KW: necropsy, lab analyses, and manuscript writing. KG and ER: animal handling and lab analyses. JH: study design, lung/kidney histopathology, morphometry, data analyses, manuscript preparation, and project funding. JP: planning, coordination, oversight, manuscript preparation/submission, and project funding. All authors contributed to the article and approved the submitted version.

## Conflict of Interest

The authors declare that the research was conducted in the absence of any commercial or financial relationships that could be construed as a potential conflict of interest.
