# Supplementary material for: Rapid Induction of Pulmonary Inflammation, Autoimmune Gene Expression, and Ectopic Lymphoid Neogenesis Following Acute Silica Exposure in Lupus-Prone Mice
Source: Front Immunol. 2021 Feb 23;12:635138. doi: 10.3389/fimmu.2021.635138 (PMC7959771; doi:10.3389/fimmu.2021.635138)
Supplement: Supplementary file 3 [file Data_Sheet_1.PDF]

Supplementary Data

Rapid induction of pulmonary inflammation, autoimmune gene expression, and ectopic lymphoid neogenesis following acute silica exposure in lupus-prone mice

Preeti S Chauhan[1,2], James G Wagner[2,3] , Abby D Benninghoff[4], Ryan P Lewandowski[3], Olivia K Favor[2,5], Kathryn A Wierenga[2,6], Kristen N. Gilley[1], Elizabeth A Ross[1], Jack R Harkema[2, 3,5], and James J Pestka[1,2,7*]

Supplementary Figure 1. NanoString autoimmune gene expression analysis reveals distinct gene expression between mice intranasally instilled with vehicle and $cSiO_2$ (2.5 mg) in lung tissue after 7, 14, 21 and 28 d PI. (A) Heatmap displays row-centered $\log_2$ counts of autoimmune-associated genes (mRNA) included in one pooled biological sample from each group. Genes were based on Euclidean distance and ward clustering. Colors are scaled per row, and red denotes high expression whereas blue low expression. Lung RNA was collected (biological samples, pooled sample from each group) and subjected to NanoString analysis using the nCounter autoimmune gene expression panel. (B) Principal component analysis (PCA) of $\log_2$ mRNA normalized counts from lung tissue of mice exposed to Veh or 2.5 mg $cSiO_2$. PC1 and PC2 shows 95% confidence interval bands. Principal component analysis shows clear clustering by Veh and $cSiO_2$. Each dot represents one biological pooled sample from each group from time course study (Blue dot = Veh sample, Red dot = $cSiO_2$ sample). Principal component 1 (PC1) separated the Veh samples (blue) from the $cSiO_2$ samples (red). Principal component 2 (PC2) separated the $cSiO_2$ samples based on d PI. Ellipses were drawn with 95% confidence.

Supplementary Figure 2. Gene Ontology (GO) enrichment analysis of significantly enriched GO term of biological processes and molecular functions for commonly upregulated genes. Bar charts depicting significantly enriched terms of (A) (biological process) BP and (B) molecular function (MF), identified by Enrichr analysis using the default parameters sorted by p-value ranking.

Supplementary Table 1 . Microsoft Excel document with output from nSolver for common upregulated differential expressed genes.

Supplementary Table 2. Microsoft Excel document with output from STRING for genes cluster.

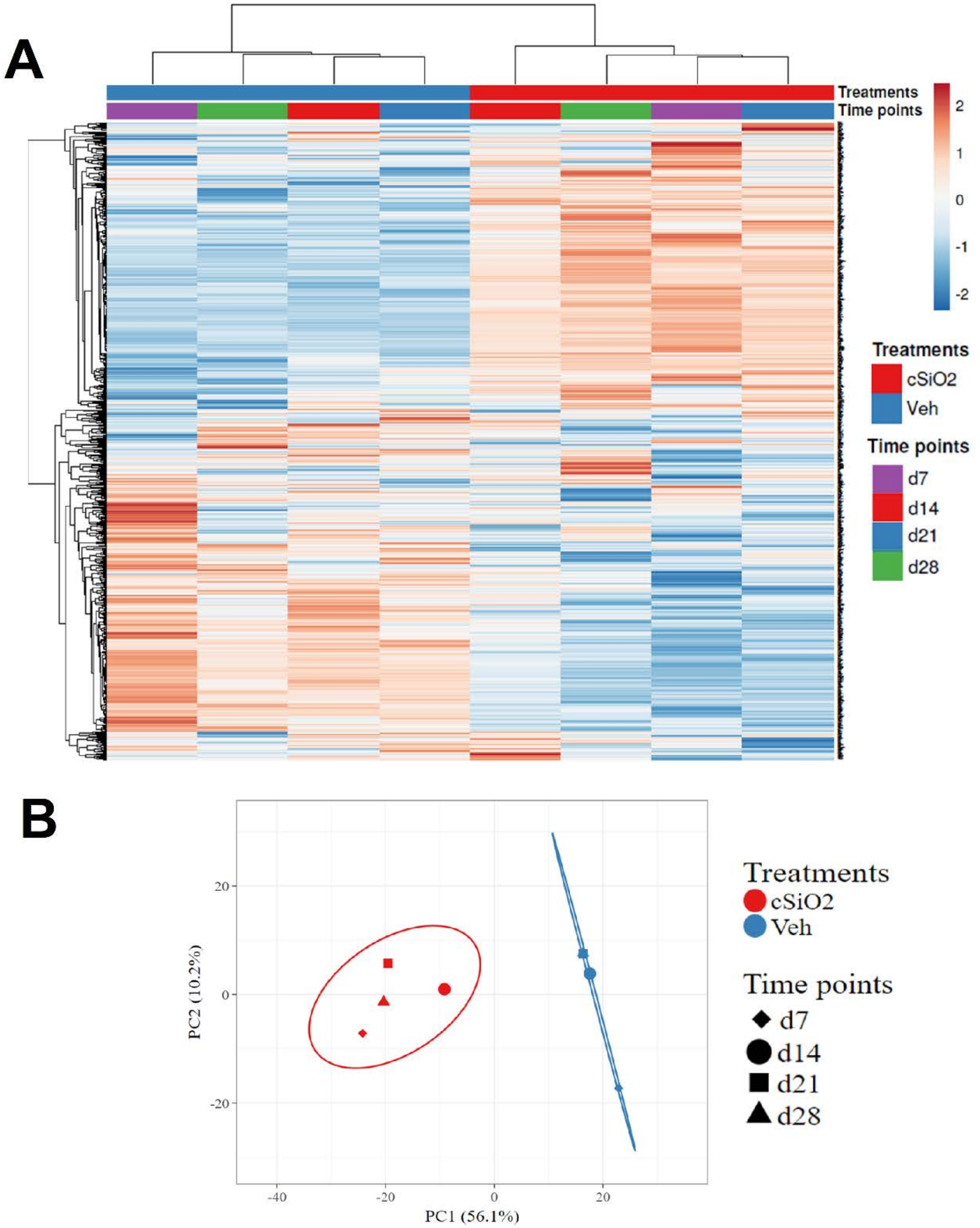

Supplementary Fig. 1

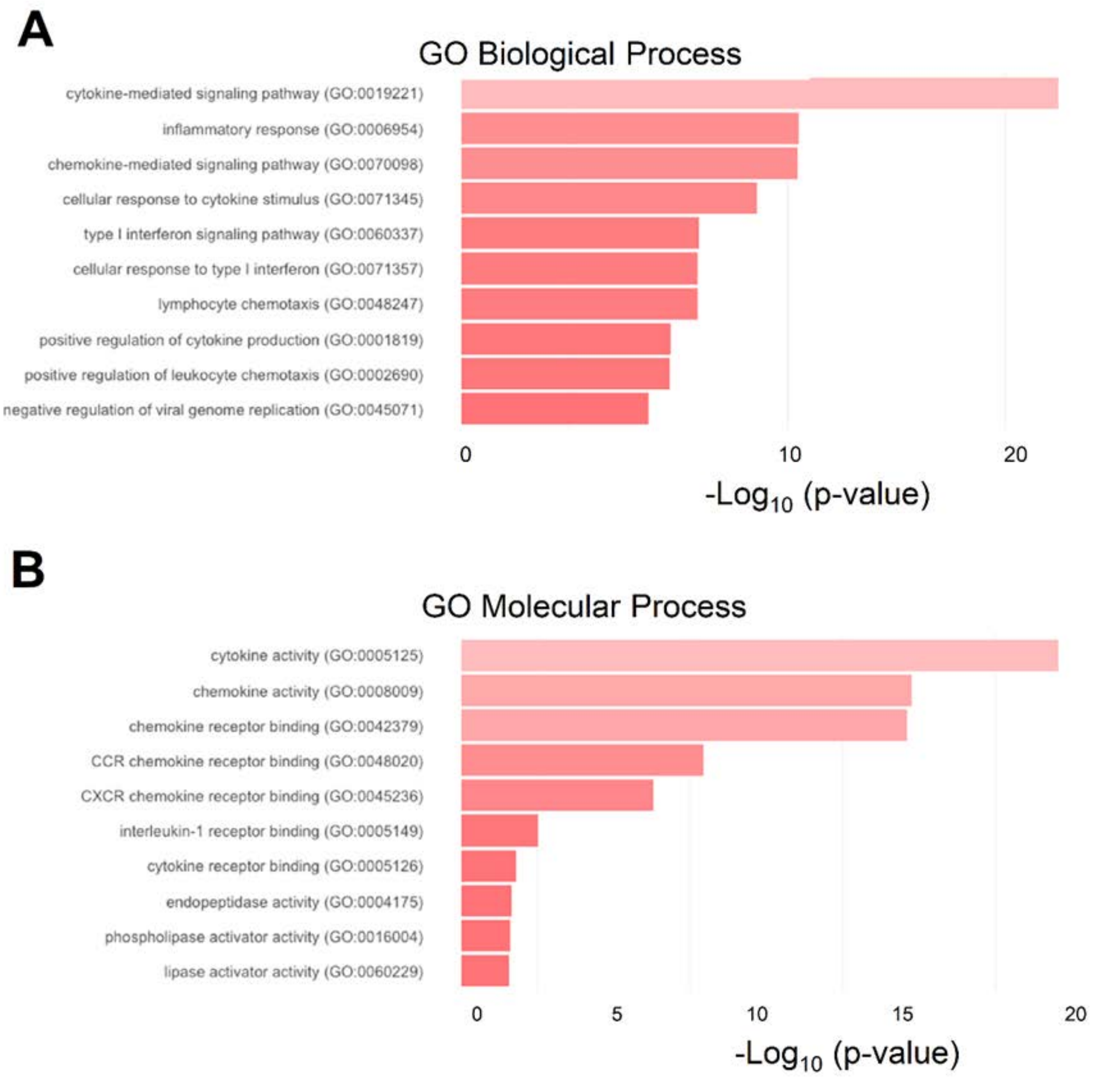

Supplementary Fig. 2
